# Complete genome sequence of *Azospirillum argentinense* A2 (DSM 2297)

**DOI:** 10.1128/mra.00161-26

**Published:** 2026-07-10

**Authors:** Kayla M. Clouse, Manuel Kleiner, Maggie R. Wagner

**Affiliations:** 1 Department of Plant Science, Pennsylvania State University 311373, University Park, Pennsylvania, USA; 2 Department of Ecology and Evolutionary Biology, University of Kansas 123984 , Lawrence, Kansas, USA; 3 Kansas Biological Survey & Center for Ecological Research, University of Kansas 4202 , Lawrence, Kansas, USA; 4 Department of Plant and Microbial Biology, North Carolina State University 242504, Raleigh, North Carolina, USA; University of Maryland School of Medicine, Baltimore, Maryland, USA

**Keywords:** plant-microbe interactions, plant growth-promoting bacteria, *Azospirillum argentinense*, nitrogen fixation, phytohormone synthesis

## Abstract

Here, we present the genome sequence of *Azospirillum argentinense* A2 (DSM 2297), which was previously identified as *Azospirillum brasilense* A2. The availability of this genome supports comparative genomic analyses and provides further species-level resolution within the genus *Azospirillum*.

## ANNOUNCEMENT

*Azospirillum argentinense* is a gram-negative, nitrogen-fixing bacterium recently described as a distinct species within the genus *Azospirillum* (1). *A. argentinense* has been widely used as a bioinoculant to promote plant growth primarily through nitrogen fixation and phytohormone production (2). Here, we present the genome sequence of *A. argentinense* A2, which was isolated from maize roots in Nigeria and previously identified as *A. brasilense* A2 (3).

*A. argentinense* A2 (DSM 2297) was obtained from DSMZ in 2023 and stored as a glycerol stock at −80°C. The strain was cultured on R2A at 30°C for 48 h. Cells were then scraped, lysed in MagMAX Microbiome Bead Tubes (Thermo Fisher Scientific), and genomic DNA extracted using the DNeasy 96 PowerSoil Pro QIAcube HT Kit (QIAGEN). DNA concentration and quality were confirmed by Qubit and TapeStation prior to short- and long-read sequencing by SeqCoast Genomics (Portsmouth, NH, USA) using the Illumina DNA Prep tagmentation kit (NextSeq2000 flow cell, 2 × 150 bp) and the Nanopore Native Barcoding Kit with size selection of unsheared DNA (PromethION 2 Solo R10.4.1 flow cell), respectively. Illumina reads were demultiplexed and trimmed with DRAGEN v4.2.7 (4), while Nanopore reads were basecalled and demultiplexed using Dorado v7.6.7 (super-accurate model). Illumina sequencing yielded 59× coverage (1,791,158 paired-end reads, mean length 148.1 bp), and Nanopore sequencing yielded 326× coverage (348,608 reads, *N*_50_ = 12,942 bp).

The genome assembly was generated using Flye v2.9.6-b1802 with an estimated genome size of 7.5 Mb and plasmid detection (5). Default parameters were used unless otherwise noted. Long-read polishing was performed with Medaka v2.1.1 (r941_min_sup_g507 model) (6), followed by short-read polishing with Polypolish v0.6.1 using Illumina reads aligned with bwa v0.7.19 (7, 8). The final assembly totaled 7,591,752 bp with 68% GC content and a *N*_50_ of 2,064,099 bp. CheckM2 v1.1.0 (9) estimated 100% completeness and 0.5% contamination, and plasmids were identified using MOB-suite (mob_recon) v3.1.0 (10). The genome contains a 2.94 Mb chromosome (68.6% GC); four chromids: AaSpA2_p1 (2.06 Mb, 68.0% GC), AaSpA2_p2 (993 kb, 68.4% GC), AaSpA2_p3 (643 kb, 69.3% GC), and AaSpA2_p4 (619 kb, 69.9% GC); a plasmid, AaSpA2_p5 (164 kb, 66.2% GC); and an accessory replicon, AaSpA2_p6 (165 kb, 63.7% GC). All contigs were circularized by Flye v2.9.6-b1802, which automatically identified and trimmed overlaps without rotating genomes. Genome annotation was performed using Prokka v1.14.6 with the default bacterial database (11), predicting 6,708 CDSs, 100 tRNAs, 1 tmRNA, and 9 rRNA operons. AaSpA2_p1 contains three complete rRNA operons and one operon lacking the 5S subunit. AaSpA2_p3 and the chromosome each contain two complete rRNA operons, and AaSpA2_p2 contains one complete rRNA operon. Multipartite genomes and incomplete rRNA operons are characteristic of *Azospirillum* species (12, 13).

Genome-wide average nucleotide identity (ANI) was calculated using FastANI v1.34 to assess species identity (14). The reference data set included all publicly available *Azospirillum* genomes previously curated (15), excluding five genomes removed by NCBI due to quality concerns (GCA_007827795.1, GCA_007827915.1, GCA_013340985.1, GCA_016622085.1, and GCA_016652835.1; 73 total genomes). Strain A2 exhibited its highest similarity to *A. argentinense*, supporting its classification within this species (Table 1).

**TABLE 1:** Species-level summary of average nucleotide identity (ANI) between strain A2 and a curated database of all publicly available *Azospirillum* genomes^*a*^

Reference species	Number of genomes	Max ANI (%)	Mean ANI (%)
*Azospirillum argentinense*	8	98.56	97.66
*Azospirillum baldaniorum*	3	95.83	95.78
*Azospirillum brasilense*	23	95.11	94.76
*Azospirillum formosense*	4	94.73	94.57
*Azospirillum tabaci*	1	94.31	94.31
*Azospirillum aestuarii*	1	94.12	94.12
*Azospirillum isscasi*	1	91.85	91.85
*Azospirillum rugosum*	2	86.03	86.01
*Azospirillum canadense*	1	85.57	85.57
*Azospirillum soli*	1	85.11	85.11
*Azospirillum agricola*	2	83.56	83.55
*Azospirillum doebereinerae*	2	83.23	83.18
*Azospirillum thermophilum*	1	83.14	83.14
*Azospirillum ramasamyi*	1	82.88	82.88
*Azospirillum melinis*	1	82.85	82.85
*Azospirillum thiophilum*	3	82.83	82.76
*Azospirillum palustre*	1	82.82	82.82
*Azospirillum lipoferum*	3	82.80	82.56
*Azospirillum endophyticum*	1	82.73	82.73
*Azospirillum humicireducens*	1	82.56	82.56
*Azospirillum oryzae*	4	82.53	82.48
*Azospirillum picis*	2	82.26	82.26
*Azospirillum griseum*	1	81.06	81.06
*Azospirillum oleiclasticum*	2	81.06	81.06
*Azospirillum cavernae*	1	81.04	81.04
*Azospirillum halopraeferens*	1	80.70	80.70
*Azospirillum fermentarium*	1	80.56	80.56

^*a*^ Maximum and mean ANI values are shown for each species.

## Data Availability

The genome sequence of *A. argentinense* A2 (DSM 2297) is available in GenBank under accession number JBTNQJ000000000. Raw Illumina and Oxford Nanopore Technologies sequencing reads are available in the Sequence Read Archive under accession numbers SRR36429349 and SRR36429347, respectively.
